# TOMM20 as a driver of cancer aggressiveness via oxidative phosphorylation, maintenance of a reduced state, and resistance to apoptosis

**DOI:** 10.1002/1878-0261.70002

**Published:** 2025-02-25

**Authors:** Ranakul Islam, Megan E. Roche, Zhao Lin, Diana Whitaker‐Menezes, Victor Diaz‐Barros, Eurico Serrano, Maria Paula Martinez Cantarin, Nancy J. Philp, Atrayee Basu Mallick, Ubaldo Martinez‐Outschoorn

**Affiliations:** ^1^ Department of Medical Oncology, Sidney Kimmel Comprehensive Cancer Center Thomas Jefferson University Philadelphia PA USA; ^2^ Division of Nephrology, Department of Medicine, Sidney Kimmel Medical College Thomas Jefferson University Philadelphia PA USA; ^3^ Department of Pathology, Anatomy & Cell Biology Thomas Jefferson University Philadelphia PA USA

**Keywords:** apoptosis, mitochondria, OXPHOS, redox, ROS, TOMM

## Abstract

Chondrosarcomas are common bone sarcomas frequently resistant to radiation and chemotherapy, with high recurrence rates, development of metastatic disease, and death. Fibrosarcomas are soft tissue sarcomas associated with poor outcomes. Translocase of outer mitochondrial membrane receptor 20 (TOMM20) is a mitochondrial receptor protein associated with cancer aggressiveness in many cancer subtypes, but the mechanisms remain poorly understood. Here, we studied the effects of TOMM20 overexpression and downregulation on the redox state, mitochondrial oxidative phosphorylation (OXPHOS), and tumor growth using fibrosarcoma and chondrosarcoma models. TOMM20 overexpression increased OXPHOS, NADH, and NADPH with reduced cellular reactive oxygen species (ROS). TOMM20 induced resistance to apoptosis, including with BCL‐2 and OXPHOS complex IV inhibitors, but with increased sensitivity to an OXPHOS complex I inhibitor. Also, TOMM20 induced cell growth and migration *in vitro* and promoted tumor growth *in vivo*. Conversely, knocking down TOMM20 using CRISPR‐Cas9 reduced cancer aggressiveness *in vivo* in both chondrosarcoma and fibrosarcoma mouse models. In conclusion, TOMM20 is a driver of cancer aggressiveness by OXPHOS, apoptosis resistance, and the maintenance of a reduced state.

## Graphical abstract

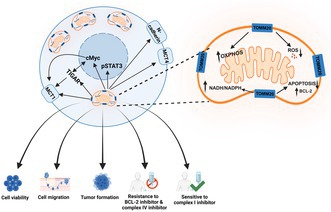

**Abbreviations**
ECAR: Extracellular acidification rate
NADH: Nicotinamide adenine dinucleotide (reduced form)
NADPH: Nicotinamide adenine dinucleotide phosphate (reduced form)
OCR: Oxygen consumption rate
OXPHOS: oxidative phosphorylation
ROS: Reactive oxygen species
SOD: superoxide dismutase
TOMM 20: Translocase of the outer mitochondrial membrane 20

## 1 Introduction

Sarcomas are cancer subtypes that originate from transformed mesenchymal cells found in connective tissues such as bone, muscle, fat, and cartilage [1, 2]. Sarcomas are broadly classified into soft tissue sarcomas and bone sarcomas [3]. Sarcomas are rare cancers in adults, accounting for approximately 1% of all cancer diagnoses, whereas in children, they represent up to 20% of all cancer cases [4]. Although most sarcomas are initially identified as localized tumors and treated with excisional surgery and radiation, metastasis can develop in up to 50% of cases within the first 5 years, depending on the tumor's location, grade, and subtype [5]. Due to the limited effectiveness of current systemic treatments, the 5‐year relative survival rate for patients with distant metastasis is <20% [3, 6].

Fibrosarcoma is a rare yet aggressive type of soft tissue sarcoma, which is characterized by the proliferation of fibroblasts with variable collagen production [7, 8, 9]. Approximately 80% of adult fibrosarcomas are high‐grade. Additionally, 25% of those initially identified as low‐grade fibrosarcomas eventually recur locally as high‐grade tumors. These tumors are frequently resistant to treatment with rates of recurrence of approximately 50% with a high risk of developing lymph node and parenchymal metastatic disease [7, 10]. Adult fibrosarcoma has poor outcomes, with survival rates below 70% at 2 years and further declining to under 55% at 5 years [11, 12].

Chondrosarcomas are bone sarcomas that predominantly affect adults [13]. The five‐year overall survival rate for grade II chondrosarcoma is approximately 75%, but overall survival drops in grade III chondrosarcomas to about 30% [3, 14, 15, 16]. Unfortunately, metastatic chondrosarcoma is incurable, resistant to chemotherapy and radiation therapy, and the median survival is less than 15 months [17, 18, 19].

There is a growing appreciation of the importance of mitochondrial oxidative phosphorylation metabolism (OXPHOS) in human cancers [20, 21, 22, 23]. Mitochondria regulate metabolism, redox homeostasis, and cell death susceptibility [23, 24, 25]. Most mitochondrial proteins are encoded by nuclear genes, synthesized in the cytosol, and guided to the mitochondria by specific signal sequences [26]. The Translocase of the Outer Membrane (TOMM) is a complex of outer mitochondrial membrane proteins and its subunit 20 (TOMM20) plays a crucial role in this process by guiding these cytoplasmic proteins into the mitochondria [27, 28, 29, 30, 31]. TOMM20 forms clusters on the mitochondrial membrane, with the density of these clusters directly correlating with mitochondrial mass, membrane potential, and metabolic activity [32]. In high‐grade human chondrosarcoma tumors, TOMM20 expression is significantly higher than in low‐grade tumors [33]. TOMM20 overexpression in human chondrosarcoma cells induces proliferation and resistance to apoptosis, chemotherapy resistance, and increases tumor growth [33]. Additionally, TOMM20 is a prognostic biomarker in chordomas, which are a rare type of bone sarcomas, and TOMM20 expression is higher in recurrent and metastatic chordomas compared to primary tumors [34]. Also, TOMM20 is highly expressed in many other human cancers and is a prognostic biomarker [35]. This highlights the potential importance of TOMM20 in cancer aggressiveness. However, the mechanisms by which TOMM20 drives cancer progression are poorly understood.

In this study, we examined the impact of TOMM20 on OXPHOS, redox state, oxidative stress, cell growth, resistance to apoptosis, migration, and tumor growth.

## 2 Methods

### 2.1 Cell culture

The mouse fibrosarcoma cell line MCA‐205 (RRID: CVCL_VR90) was purchased from Sigma (St. Louis, MO, USA) and cultured in RPMI media containing 4.5 g·L^−1^ glucose, 1 mm pyruvate, 10% fetal bovine serum (FBS), 100 units·mL^−1^ penicillin, 100 units·mL^−1^ streptomycin. 1× nonessential amino acids, 1× β‐mercaptoethanol at 37 °C, 5% CO_2_. Human chondrosarcoma CH2879 (RRID: CVCL_9921) and L2975 cells (RRID: CVCL_D706) were obtained from Professor Judith Bovee (Leiden University Medical Center, the Netherlands) and Professor Antonio Llombart (Universidad de Valencia, Spain) and cultured in RPMI media containing 10% FBS. Mouse MOC‐1 (RRID: CVCL_E1HL) cells were obtained from Professor Ravindra Uppaluri (Dana Farber Cancer Institute) and cultured in a 2 : 1 mixture of HyClone™ Iscove's Modified Dulbecco's Medium (IMDM; GE Healthcare Life Sciences, South Logan, UT, USA) and Hyclone™ Ham's Nutrient Mixture F12 (GE Healthcare Life Sciences). The culture medium also contained 10% FBS (Life Technologies, Gaithersburg, MD, USA), 1% penicillin/streptomycin (Life Technologies), 5 ng·mL^−1^ epidermal growth factor (Sigma), 40 ng·mL^−1^ hydrocortisone (Sigma), and 5 ng·mL^−1^ insulin (Sigma). In 2024, we submitted these cell lines to ATCC for verification using short tandem repeat (STR) profiling and cross‐referenced with the Cellosaurus STR database. All cell lines showed a 100% match with the Cellosaurus database. All experiments were conducted using mycoplasma‐free cells.

### 2.2 Lentiviral plasmids and virus infection

For overexpression of TOMM20 in L2975, CH2879, and MCA205 cells, lentiviral plasmids HA‐TOMM20 (EX‐G0283‐Lv120) and HA‐control (EX‐NEG‐Lv120) were used. In addition, TOMM20 plasmid EX‐Mm09496‐Lv207 and negative control Ex‐Neg‐Lv207 were used for MOC‐1 cells. Plasmids were obtained from GeneCopoeia (Rockville, MD, USA), and the lentiviral particles were prepared following the manufacturer's protocol. Cells were cultured at 2.5 × 10^5^ cells per well in 2 mL RPMI complete media, and 24‐h postculture, 1 mL of growth media was replaced with 1 mL of virus‐containing media. Later, 5 μg·mL^−1^ polybrene was added to the media. Following infection, the cells were treated with puromycin (2.5 μg·mL^−1^) for 5 days for selection.

To knock down TOMM20 in MCA‐205, 2.5 × 10^5^ MCA‐205 cells were transduced with LentiCas9‐G418 lentivirus, and 5 μg·mL^−1^ polybrene was added to the medium. Gentamicin was added after 24 h to select stable transductants. Cas‐9 expressing cells were transduced with lentivirus containing plasmids with three murine target guide RNAs MCP303420‐LvSG03‐3‐10‐a: GGAGAGAGCTGGGCTTTCCA, MCP303420‐LvSG03‐3‐10‐b: GTCTGCAGGTGACTACGAGA, and MCP303420‐LvSG03‐3‐10‐c: CAAGCTTCCGACCATTAGTC, which were purchased from Genecopoeia (Rockville, MD, USA) and packaged into HEK‐293T cells. Single clones were identified using 96‐well plates. TOMM20 knockdown was confirmed by western blot analysis. Two clones were selected and named KD#1 and KD#2. To knock down TOMM20 in human CH2879 cells, we used HCP317434‐LvSG03‐3‐10, which also had 3 × sgRNA lentiviral expression clones. We used scrambled sgRNA pCRISPR‐LvSG03 as a control.

### 2.3 RNA‐seq analysis

L2975 cells with TOMM20 overexpression and controls were cultured in RPMI media containing 4.5 g·L^−1^ glucose, 1 mm pyruvate, 10% FBS, 100 units·mL^−1^ penicillin, and 100 units·mL^−1^ streptomycin at 37 °C, 5% CO_2_. Total RNA was isolated with a Qiagen kit per manufacturer instructions. The whole transcriptome expression was performed at Sidney Kimmel Cancer Center of Thomas Jefferson University's Cancer Genomics facility.

### 2.4 Cell growth/viability assay

Cells were grown in 12‐well plates, and live cells were counted for 4–6 consecutive days using Countess™ Cell Counting Chamber Slides (Fisher Scientific, Hampton, NH, USA) and trypan blue, 0.4% (Fisher Scientific).

### 2.5 Transwell migration assay

2.5 × 10^5^–1 × 10^6^ lentiviral containing stable cells were seeded into RPMI media containing NuSerum and added to 8.0‐μm pore transwell chambers (Corning, Kennebunk, ME, USA). The transwells were placed in 24‐well plates containing media with 10% FBS‐containing media. Cells were incubated at 37 °C and 5% CO_2_. After 24 h of incubation, a cotton swab was used to remove nonmigrated cells, and migrated cells were fixed with 70% alcohol, stained with crystal violet (0.1%), and photographed under a phase microscope with an iPhone 13 pro using a LabCam ocular adapter (LabCam, New York, NY, USA). ImageJ was used to count cell numbers.

### 2.6 Measurement of oxygen consumption rate (OCR) from cells and microtissues

Seahorse Bioscience XF24 Extracellular Flux Analyzer was used for the assay. Approximately 30 000 MCA‐205 cells were seeded into Seahorse XF24 cell culture plate with 100 μL of RPMI 1640 media containing 10% FBS, 100 units·mL^−1^ penicillin–streptomycin. After one hour, an additional 200 μL of growth medium was added for a final volume of 300 μL per well. The next day, cells were incubated in nonbuffered RPMI 1640 media containing 5 mm glucose for 2 h. Measurements were taken under three conditions: baseline, after injection of 2 mm glutamine or injection of 10 mm lactate, and after injection of Antimycin A and Rotenone. For experiments evaluating maximal respiration, OCR was measured at baseline, after injection of 2.0 μm FCCP, and after injection of 0.5 μm Antimycin A and Rotenone. OCR measurements were normalized to cell number.

To measure the OCR and extracellular acidification (ECAR), which refers to the decrease in pH of the extracellular environment surrounding cells from tumor microtissues, we followed the previously described protocol [36]. The tumors were sliced into consistent 1 mm pieces and placed in the middle of the islet capture microplate with 100 μL of unbuffered RPMI media. Then, an additional 400 μL RPMI media was added to the microplate for a final well volume of 500 μL. The microtissues were kept for 1 h in nonbuffered seahorse media in a CO_2_‐free incubator. Total protein was used to normalize both OCR and ECAR.

### 2.7 Mito‐Fuel dependency

In examining mitochondrial fuel oxidation pathways, the Mito‐Fuel assay from Agilent Technologies (Santa Clara, CA, USA) was utilized using the company's protocol. In the Mito‐Fuel Flex test, three inhibitors were used to block the entry of three essential metabolic fuels—pyruvate, fatty acids, and glutamine—into the mitochondria. This blockade was achieved using three distinct inhibitors: first, mitochondrial pyruvate carrier was inhibited by 2 mm of UK5099, second, carnitine palmitoyl transferase 1A was inhibited by 4 mm of etomoxir (ETO), and then, glutaminase was inhibited by 3 mm of BPTES. The oxygen consumption rate (OCR) was initially measured to determine the baseline. After the baseline OCR reading, sequential inhibitor injections were performed and are referred to as Treatment 1 (target inhibitor) and Treatment 2 (other two inhibitors) with subsequent monitoring of the OCR after each treatment. Protein concentration was used to normalize the oxygen consumption rate (OCR). The fuel dependency, capacity, and flexibility for each fuel were calculated as follows:

$$\text{Dependency}\left(\%\right)=\begin{array}{l} \left[\left(\text{Baseline}\;\mathrm{OCR}–\text{Target inhibitor}\;\mathrm{OCR}\right)/\;\left(\text{Baseline}\;\mathrm{OCR}–\mathrm{all}\;\text{inhibitor}\;\mathrm{OCR}\right)\right]\;\times 100 \end{array}$$

$$\text{Capacity}\left(\%\right)=\begin{array}{l} \left[1-\left(\text{Baseline}\;\mathrm{OCR}–\text{other}\;2\;\text{inhibitors}\;\mathrm{OCR}\right)/\;\left(\text{Baseline}\;\mathrm{OCR}–\mathrm{all}\;\text{inhibitor}\;\mathrm{OCR}\right)\right]\\ \times 100 \end{array}$$

$$\text{Flexibility}\left(\%\right)\;=\text{difference between capacity}\left(\%\right)\;\text{and dependency}\left(\%\right)$$

### 2.8 *In vivo* tumor growth assay and PD1 antibody injection

C57BL/6 and nude mice were acquired from the Jackson Laboratory (Farmington, CT, USA). MCA‐205 and MOC‐1 cells were injected into syngeneic C57BL/6 mice, and nude mice were used for human CH2879 cells. 1× 10^6^ cells in 100 μL of PBS were subcutaneously injected into both flanks. Each group had at least eight mice injected bilaterally, receiving at least 16 injections. Tumor sizes were measured every other day. Formula *V* = (*X* ^2^ *Y*)/2 was used to calculate the volume of the tumor where *V* = Volume (mm^3^), *X* = width (mm^2^), and *Y* = length (mm). Tumors were collected, weighed, flash frozen for western blot, and fixed in formalin for immunohistochemistry. The protocol for the animal study at Thomas Jefferson University received approval from the Institutional Animal Care and Use Committee (IACUC). All procedures adhered to the guidelines outlined in the Guide for the Care and Use of Laboratory Animals.

The effect of the PD‐1 antibody was investigated in mice receiving TOMM20 overexpressed MCA‐205 cells or control cells. Mice were first injected with MCA‐205 cells and later received intraperitoneal injections with anti‐mouse PD‐1 (CD279) antibody (# BE0146, BioXCell, Hayward, CA, USA). Each mouse received a dose of 200 μg of antibody in 200 μL of pH 6.5 Dilution Buffer (BioXcell # IP0065). The injections were performed on days 1, 5, and 8, and external measurements for tumor size were obtained to follow tumor growth.

### 2.9 Immunoblotting assay

RIPA buffer (Sigma) containing 100 μL protease and phosphatase inhibitors (Thermo Scientific, Rockford, IL, USA) per 10 mL, was used for cell lysis. 15–20 μg total protein was used for each loading and separated by 2–10% premade gels (Fisher). Subsequently, the proteins were transferred to PVDF/nitrocellulose membrane and blocked with 5% nonfat milk. The primary antibodies are listed in Table 1 and incubated for 12–16 h at 4 °C in a solution containing 5% bovine serum albumin (BSA, Sigma). Goat anti‐mouse (#R1005) and goat anti‐rabbit (#R1006) from Kindle Bioscience (Kindle Biosciences, Greenwich, CT, USA) were used as secondary antibodies for 2 h in 5% nonfat milk at room temperature. Protein bands were visualized and captured using the KwikQuant Western Blot Detection Kit (Kindle Biosciences) and the ChemiDoc Touch Imaging System.

**Table 1:** Primary antibodies and their dilutions.

Protein	Company	Reference	Dilution
A1/Bfl‐1	CST	14 093	1:1000
β‐actin	Sigma	A2228	1:3000
BAD	BD Bioscience	610 392	1:1000
BAX	Sigma	06‐499	1:1000
BCL2	Abcam	ab181858	1:1000
BCL‐xL	CST	2764	1:1000
BCL‐w	CST	2724	1:1000
Caspase 3	CST	9662	1:1000
Cleaved Caspase 3	CST	9661	1:1000
c‐MYC	CST	T5605	1:1000
MCL1	CST	94 296	1:1000
MCT1	Santa Cruz Biotechnology	sc‐365 501	1:1000
MCT4	Proteintech	22 787‐1‐AP	1:2000
N‐Cadherin	CST	13 116	1:1000
PDL‐1	CST	#13684	1:1000
Phosphorylated STAT3 Tyrosine 705	CST	9145	1:1000
TIGAR	CST	ab31910	1:1000
TOMM20	CST	42 406	1:1000
TOMM70	Sigma	HPA014589	1:1000
TOMM40	CST	55 959	1:1000
TOMM22	Proteintech	11 278‐1‐AP	1:1000
Total OXPHOS	Abcam	ab110411	1:1000
Catalase	CST	14 097	1:1000
SOD1	CST	37 385	1:1000
SOD2	CST	13 141	1:1000

### 2.10 NAD/NADH and NADP/NADPH ratio

EnzyChrom™ NAD/NADH Assay kit (Cat # E2ND‐100; BioAssay Systems, Hayward, CA, USA) and EnzyChrom™ NADP/NADPH Assay kit (# E2NP‐100; BioAssay Systems) were used to measure NAD/NADH ratio and NADP/NADPH ratio in the cells using manufacturer's instructions. Briefly, 1 × 10^5^ – 5 × 10^5^ cells were used, homogenized in extraction buffers, and heated for 5 min at 60 °C. Subsequently, 20 μL of assay buffer and 100 μL of the opposite buffer were used to stop the reaction. 80 μL of working reagent was added to 96 plates containing 40 μL of sample and standard. Absorbance was measured at 0 and 15 min for NAD/NADH and at 0 and 30 min for NADP/NADPH OD_565_ at room temperature. The concentration and ratio were calculated using the manufacturer's equation.

### 2.11 Flow cytometry

Annexin‐V‐APC (#A35110; Life Technologies) and propidium iodide (PI) were utilized to detect apoptotic cells and live cells as previously described [37]. Briefly, 5 × 10^4^–1 × 10^5^ cells were seeded into 12‐well plates. The next day, complete media was replaced with 10% NuSerum media. The cells were treated with the drug for 24 h before apoptosis was evaluated. The media, PBS wash, and cells were harvested and stained with a master mix containing the annexin V‐APC conjugate (2 μL) and PI (1 μL) in 500 μL buffer or V‐APC conjugate (2 μL) DAPI and (0.02 μL) in 500 μL. The cells were stained in the dark at room temperature for 15 min.

To measure cellular oxidative stress, CellROX™ Deep Red Reagent (# C10422; Life Technologies) was used. 5 × 10^4^ cells were plated into 12‐well plates 72 h before the experiment. The next day, the complete media was replaced with 10% NuSerum‐containing media. 2 μL of CellROX™ Deep Red Reagent was used per 1 mL media, and the cells were incubated for 30 min at 37C in the dark. Cells were collected, resuspended into 400 μL of PBS, and analyzed using flow cytometry. We used unstained controls to determine the negative population.

### 2.12 TUNEL assay and quantification of apoptotic cells in mouse tumors

Four‐micron paraffin sections were deparaffinized, rehydrated, and treated with 20 μg·mL^−1^ Proteinase K in PBS for 20 min. Following PBS washes, endogenous peroxidase activity was blocked with 3% H_2_O_2_ for 15 min and washed with ddH20 2 × 5 min. Sections were incubated with ApopTag TdT enzyme (S7107; Millipore‐Sigma, St. Louis, MO, USA) and reaction buffer (S7105; Millipore‐Sigma) at a ratio of 5:70:25 TDT/Rxn/ddH20 for 30 min at 37C. After washing 2× PBS for 5 min each, sections were treated with anti‐digoxigenin‐POD (11 207 733 910; Sigma) for half an hour at room temperature. Cells positive for TUNEL were visualized using a DAB substrate kit (CST; Cell Signaling Technology, Danvers, MA, USA). Apoptotic nuclei were calculated utilizing Aperio software (Leica Biosystems, Nußloch, Germany). Digital images were obtained with Leica and Aperio slide scanners under 320× magnification with a mean scan time of 2 min (compression quality 70). TUNEL‐positive cells were identified, and values for the percentage of 3+ nuclei were generated using a nuclear algorithm. Multiple viable tumor areas, free of necrosis, were evaluated, encompassing 80–85% of the total tumor area. Four tumors from each group were examined. Statistical significance was determined with Student's *t*‐test.

### 2.13 Immunofluorescence labeling of cultured cells

Cells were cultured in 12‐well plates for 24 h, fixed with 4% paraformaldehyde, followed by methanol permeabilization, and then blocked with 5% BSA in PBS. Rabbit anti‐HA antibody (#3724; Cell Signaling Technology) was used to detect HA‐tagged TOMM20, and mouse anti‐HSP60 (#NBP2‐32973; Novus Biologicals, Centennial, CO) was used to label mitochondria. Secondary antibodies were anti‐rabbit Alexa Fluor 568 (Thermo Scientific, A10042) and anti‐mouse Alexa Fluor 488 (Thermo Scientific, A21202). DAPI was used to stain nuclei, and slides were mounted using Prolong Gold anti‐fade Reagent (Thermo Scientific, P36934). Images were obtained with a 40× oil objective on a Nikon A1 confocal system. imagej software was used to measure the staining intensity after separation into red, green, and blue (RGB) components. The specific channel that corresponded to the desired stain was selected for analysis. The intensity of this stain in the chosen areas was then quantified and normalized to the number of nuclei present in those areas.

### 2.14 CD8 immunohistochemistry and quantification

Four‐micron paraffin sections of five tumors from each group were stained with CD8 antibody using a 3‐step avidin–biotin horseradish peroxidase method. Briefly, sections were deparaffinized, rehydrated, and antigen retrieval was performed using 10 mm sodium citrate, pH 6.0 for 10 min using an electric pressure cooker. After cooling, sections were blocked with 3% hydrogen peroxide followed by blocking for endogenous biotin with an Avidin Biotin kit (#AB972H; Biocare Medical, Pacheco, CA, USA) and incubated overnight at 4C with 10% normal goat serum. The next day, sections were incubated with anti‐CD8 antibody (#98941; Cell Signaling Technology) for one hour at room temperature. Biotinylated anti‐rabbit secondary antibody and ABC peroxidase complex (Vector Labs, Newark, CA, USA) were for 30 min each, and positive cells were detected using the SignalStain DAB substrate kit (#8509; Cell Signaling Technology).

CD8‐positive cells were quantified using qupath v.0.5.1 [38] using the positive cell detection algorithm. For each tumor section, 5–8 areas were analyzed, encompassing 85–90% of the tumor, and the percent positive CD8 cells for each area was generated for a total of 31 separate areas analyzed for each group. Significance was evaluated using Student's *t*‐test.

### 2.15 Statistical analysis

Statistical analysis was conducted using Excel, GraphPad Prism, and R. The DESeq2 and DOSE software packages were used to identify differential expressed gene and pathway analysis, respectively. Data were represented as mean ± standard error. T‐tests were used to compare the two groups, and ANOVA was used to compare the results among more than two groups. Our study defined a 5% level (*P* < 0.05) as statistically significant. Additionally, a *P*‐value of <0.1, although not meeting the strict threshold for significance, indicated a trend worth noting.

## 3 Results

### 3.1 Upregulation of TOMM20 enhanced expression of drivers of cancer aggressiveness, cell migration, and cell viability in fibrosarcoma cells

**Fig. 1:**
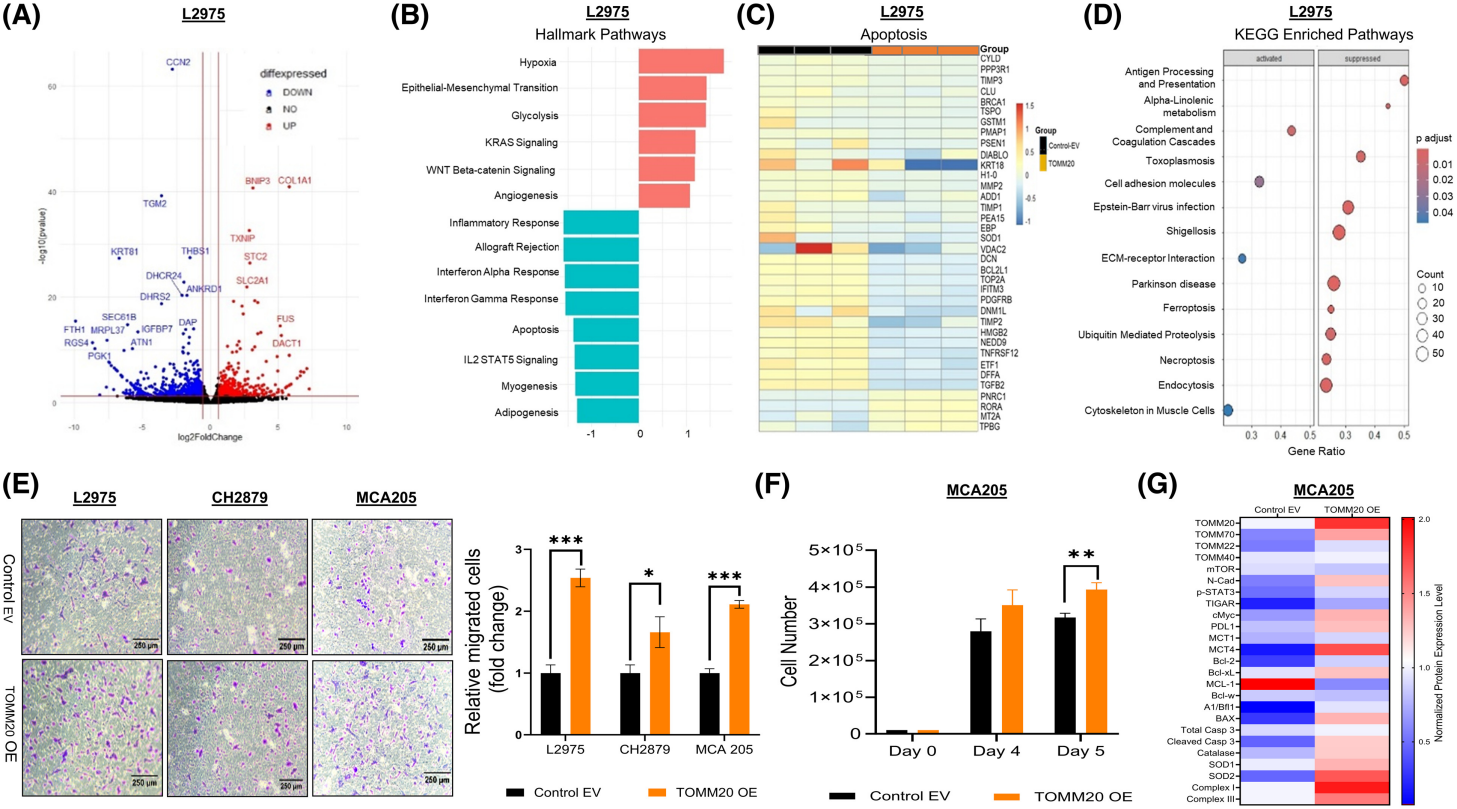
Impact of TOMM20 on cancer aggressiveness. (A–D) RNA‐seq data for L2975‐TOMM20 overexpression versus control (EV) (*N* = 3). (A) Volcano plot of the differentially expressed genes. (B) Hallmarks pathways using gene set enrichment analysis (GSEA) (C) Heatmap plots of the apoptosis pathway. (D) Kyoto Encyclopedia of Genes and Genomes (KEGG) pathways are activated or suppressed with TOMM20 overexpression in L2975 cells. (E) Cell migration assays comparing control to TOMM20 overexpression in L2975, CH2879, and MCA‐205 cells using the Boyden chamber (8 μm). (F) Cell viability assay comparing control to TOMM20 overexpression in MCA‐205. Cells were counted using trypan blue and a hemocytometer under a microscope. (G) A heatmap shows the quantification of the western blot in MCA‐205 cells and compares TOMM20 overexpression to EV control. Data are represented as mean ± SE. *P < 0.05, **P < 0.01, and ***P < 0.001 compared to the control group.

TOMM20 (Translocase of Outer Mitochondrial Membrane 20) is a mitochondrial receptor protein essential for importing mitochondrial precursor proteins [39]. Previous studies in our laboratory identified that high‐grade human chondrosarcoma tumors have high expression of TOMM20, and overexpression of TOMM20 in the human chondrosarcoma cell lines L2975 and CH2879 increased cell proliferation and tumor growth [33]. Hence, to further explore molecular biological pathways altered due to TOMM20 enrichment, we performed whole transcriptome RNA‐seq with chondrosarcoma L2975‐control and L2975‐TOMM20 overexpressing cells (Fig. 1A–D; Figs S1, S2). Figure S1A illustrates the verification of TOMM20 overexpression as identified in the RNA‐seq data. DESeq2 identified 592 genes that were downregulated and 350 that were upregulated with TOMM20 overexpression compared to control. A volcano plot, as seen in Fig. 1, displays the most differentially expressed genes at *P* < 0.05 levels (Fig. 1A). Gene set enrichment analysis (GSEA) for hallmark pathways (Fig. 1B) and reactome pathways (Fig. S1B) revealed that the apoptosis gene set was downregulated with the most differentially expressed genes shown in Fig. 1C. KEGG pathway analyses revealed that ferroptosis, ubiquitin‐mediated proteolysis, and necroptosis pathways were downregulated, while the ECM‐receptor interaction pathway was activated (Fig. 1D; Fig. S2A–D). Then, we assessed if migration was modulated by TOMM20 and determined that L2975 and CH2879 cells with TOMM20 overexpression increased migration (Fig. 1E). To determine whether TOMM20 can play a role in fibrosarcoma aggressiveness, we stably overexpressed TOMM20 (TOMM20 OE) and empty vector (Control EV) as a control in mouse fibrosarcoma cells MCA‐205 and verified TOMM20 overexpression in the mitochondria using immunofluorescence by labeling mitochondria with HSP‐60 and HA‐tagged TOMM20 with anti‐HA antibody and by western blot analysis (Fig. S3A,B). Using MCA‐205 cells, we found that increased TOMM20 led to increased cell migration (Fig. 1E) and cell viability (Fig. 1F).

To investigate potential factors contributing to increased cancer invasiveness, we assessed phosphorylated STAT3 and N‐cadherin levels (Fig. 1G; Fig. S3C). Cells with increased TOMM20 levels showed a rise in N‐cadherin and phospho‐STAT3 compared to control EV cells. Drivers of mitochondrial metabolism, c‐MYC, and TP53‐inducible glycolysis and apoptosis regulator (TIGAR) were upregulated in cells with TOMM20 overexpression compared to the control EV. Additionally, among the five known BCL‐2 family members with apoptosis inhibitory effects (BCL‐xL, BCL‐2, BCL‐w, MCL1, and A1/Bfl1), three members—BCL‐2, BCL‐xL, and A1/Bfl1—were upregulated in TOMM20‐overexpressing cells. Conversely, MCL1 was downregulated, and BCL‐w levels were similar to those in control EV. Also, the proapoptotic BH3‐only sensor BAX was upregulated in TOMM20‐overexpressing cells (Fig. 1G; Fig. S3C).

Notably, TOMM20 overexpression resulted in increased protein expression of TOMM22 and TOMM70, both of which serve as additional receptors on the mitochondrial outer membrane, while levels of the mitochondrial outer membrane pore protein TOMM40 remained unchanged. Furthermore, TOMM20‐overexpressing cells showed elevated expression of antioxidant enzymes, including catalase, SOD1, and SOD2 (Figs 1G, S3C). In these cells, there was also an increase in the mitochondrial OXPHOS subunits of complex I (Figs 1G, S3C). Moreover, monocarboxylate transporters MCT1 and MCT4 were upregulated in TOMM20‐overexpressing fibrosarcoma cells (Figs 1G, S3C). Both caspase 3 and its activated, cleaved form were upregulated in these cells. Additionally, we discovered that the immune checkpoint protein programmed death ligand 1 (PD‐L1) was upregulated in TOMM20‐overexpressing cells, which can promote tumor immune evasion [40, 41]. Interestingly, the level of mTOR did not significantly change, which is considered to shift metabolism to aerobic glycolysis by upregulating glycolytic enzymes [42], increasing glucose transporters (e.g., GLUT1), activating HIF‐1a [43], and increasing activity of the rate‐limiting factor of glycolysis, PKM2 [44]. In sum, TOMM20 overexpression increased cell viability, migration, and the expression of drivers of cancer aggressiveness in MCA‐205 cells.

### 3.2 TOMM20 promoted OXPHOS with increased NADH and NADPH and decreased ROS levels in fibrosarcoma cells

**Fig. 2:**
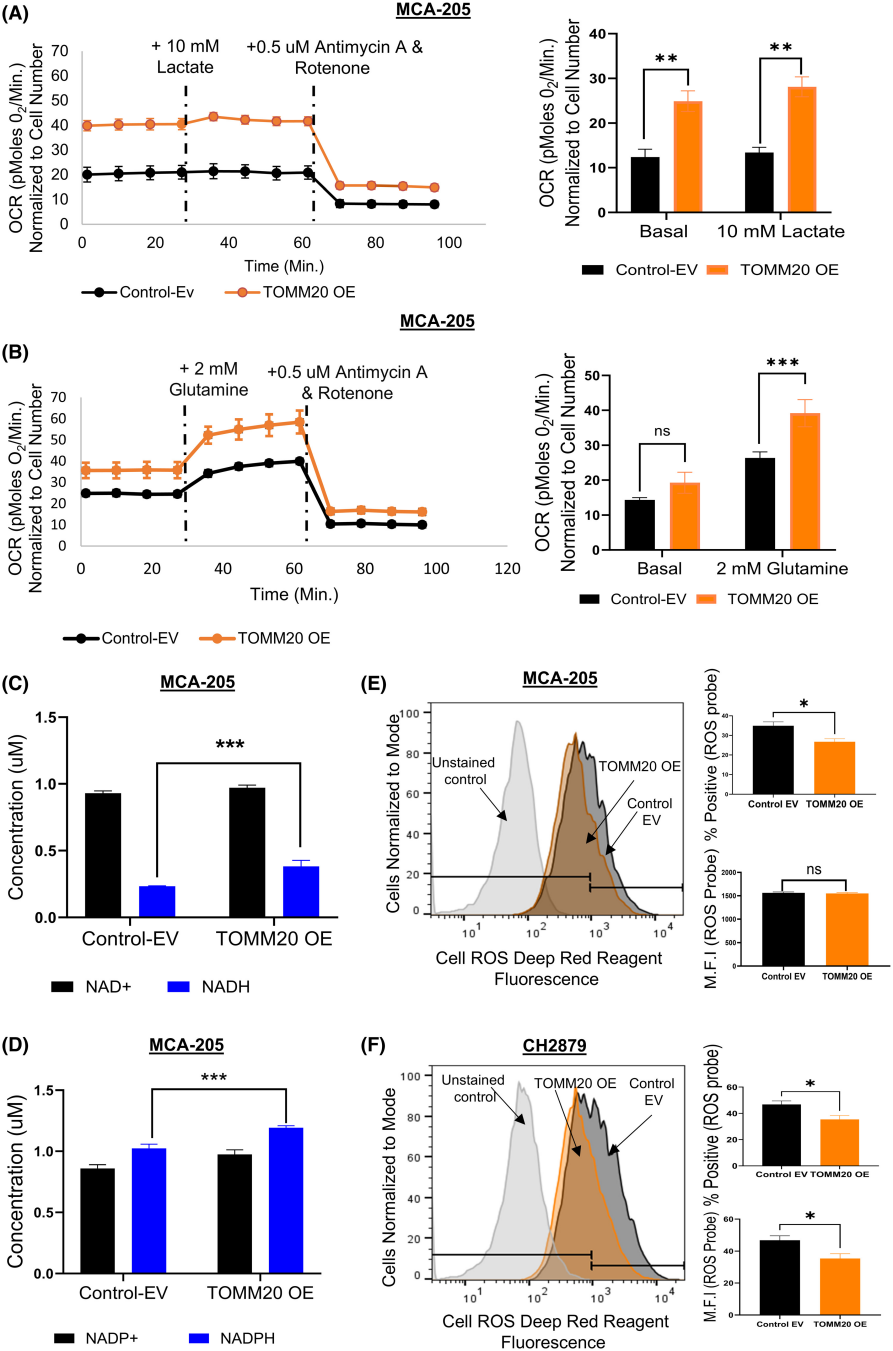
Impact of TOMM20 overexpression on cellular metabolism and ROS levels. (A, B) Oxygen consumption rate (OCR) was calculated in control MCA‐205 cells (*black bar*), and with TOMM20 overexpression (orange bar), OCR values were normalized to cell number. Cells were cultured under control media, supplemented with 2 mm glutamine, and control media with 10 mm lactate (*N* = 6). (C) NAD+ (*black bar*) and NADH concentration (*blue bar*) in control vs. with TOMM20 overexpression MCA‐205 cells (*N* = 4) (D) NADP+ (*black bar*) and NADPH concentration (blue bar) in control vs. with TOMM20 overexpression MCA‐205 cells (*N* = 4) (E) The percentage of cells positive and mean fluorescent intensity (M.F.I) for red oxidative stress reagent were measured with flow cytometry using cell ROS deep reagent in control vs. with TOMM20 overexpression MCA‐205 cells (*N* = 4) (F) The percentage of cells positive and mean fluorescent intensity (M.F.I) for red oxidative stress reagent were measured with flow cytometry in control vs. with TOMM20 overexpression CH2879 cells (*N* = 3). Two‐tailed Student's *t*‐test between groups (A–F); data represented as mean ± SE. *P < 0.05, **P < 0.01, and ***P < 0.001 compared to the control group.

Next, we aimed to investigate the influence of TOMM20, along with exposure to lactate and glutamine, on mitochondrial oxidative phosphorylation (OXPHOS) metabolism by measuring the oxygen consumption rates (OCR) in both TOMM20 overexpressing or control EV fibrosarcoma cells. We found that the baseline OCR (Fig. 2A) and the OCR after the addition of 10 mm lactate (Fig. 2A) or 2 mm glutamine (Fig. 2B) were markedly increased in the TOMM20‐overexpressing MCA‐205 cells compared to the control (*P* < 0.01). We also utilized mitochondrial uncoupler FCCP (carbonyl cyanide‐p‐trifluoromethoxyphenylhydrazone) to determine maximal respiration. We determined that TOMM20 overexpressing cells have higher maximal respiration and spare capacity (Fig. S4A). To better understand the roles of glucose, fatty acids, and glutamine in powering mitochondrial metabolism, we measured the OCR using Mitochondrial Fuel Screening (MFS), which involves three different inhibitors to shut down the catabolic pathways of each fuel type. This MFS assay showed that cells with TOMM20 overexpression had increased glutamine dependency at 29.1%, compared to 21.6% in the control cells (Fig. S4B). This result suggests that TOMM20‐overexpressing cells can utilize glutamine for their energy needs better than control cells, which is consistent with the OCR results in the presence of glutamine (Fig. 2B).

Actively proliferating cancer cells produce large amounts of reactive oxygen species (ROS), and to protect themselves from the deleterious effects of ROS, these cells require reduced NADH [45], and NADPH [46]. Consequently, we aimed to examine the NADH and NADPH levels in these fibrosarcoma cells. Cells overexpressing TOMM20 had 65% higher NADH levels than the control (Fig. 2C), resulting in a more than 30% decrease in the NAD+/NADH ratio (Fig. S4C). Additionally, TOMM20‐overexpressing cells increased NADPH production by 20% compared to controls (Fig. 2D), although there was only a trend toward a decreased NADP+/NADPH ratio (*P* = 0.09) (Fig. S4D). The observed increase in NADPH suggests an enhanced antioxidant defense or upregulation of NADPH‐generating pathways, such as the pentose phosphate pathway.

Also, we measured ROS levels using flow cytometry in MCA‐205 and CH2879 cells. We found that TOMM20 overexpression led to reduced levels of the red fluorescent fluorochrome indicative of oxidative stress in MCA‐205 and CH2879 cells (Fig. 2E,F). In conclusion, the overexpression of TOMM20 in MCA 205 cells led to an increase in reduced equivalents NADH and NADPH, enhanced oxidative phosphorylation (OXPHOS), and a reduction in reactive oxygen species (ROS).

### 3.3 Upregulation of TOMM20 induced larger tumors, enhanced the expression of drivers of cancer aggressiveness, and reduced tumor apoptosis

**Fig. 3:**
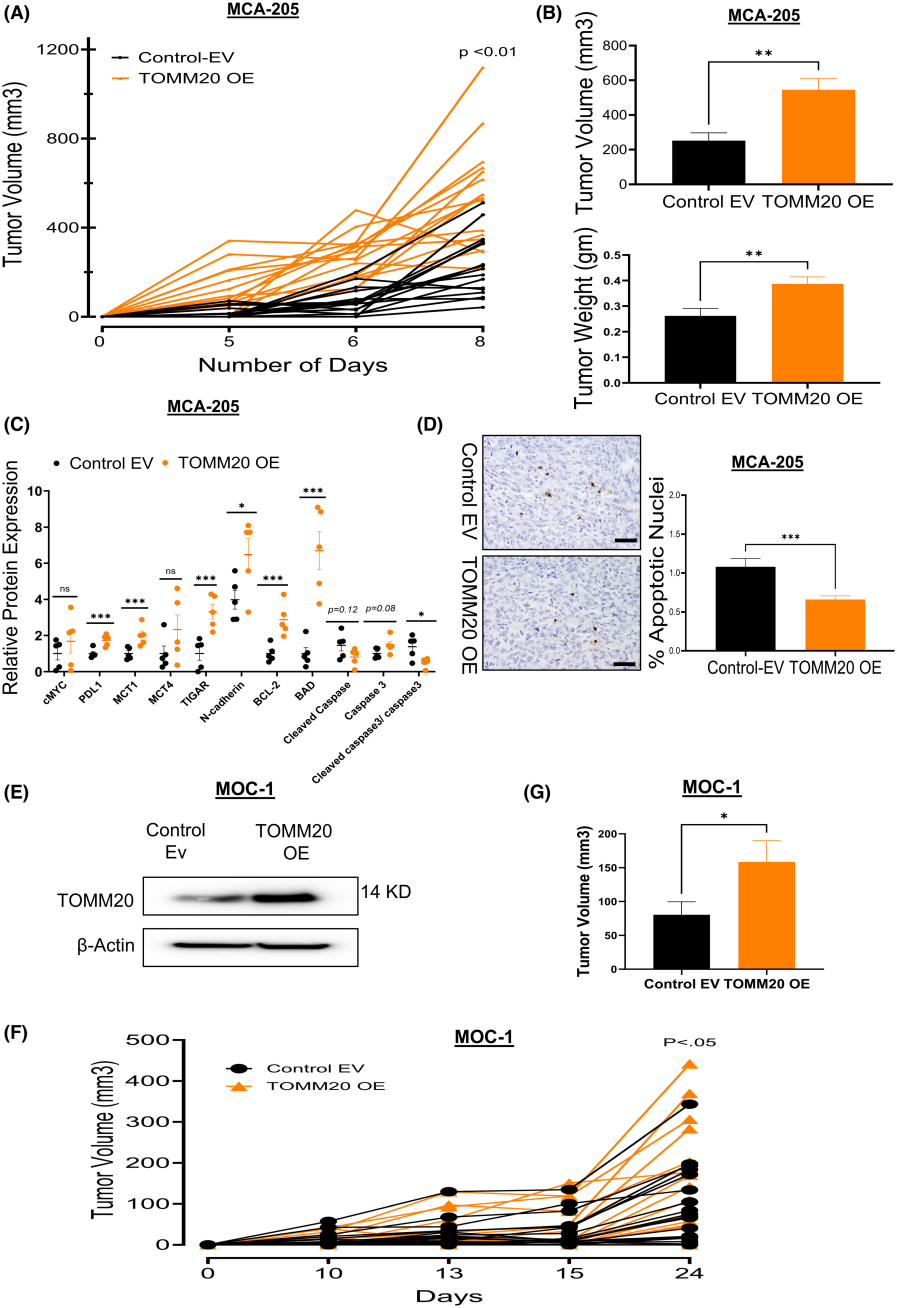
TOMM20 overexpression increases tumor growth, the drivers of cancer aggressiveness, and decreases apoptosis in fibrosarcoma tumor tissues. (A)The line graphs show individual tumor volume growth from the initial detection of tumors to the study's endpoint after injection of control EV vs. TOMM20 overexpressed MCA‐205 cells into the flank of mice (*N* = 16). (B) Tumor volume, weight, and two‐tailed Student's significant test upon tumor collection (*N* = 16). (C) The scatter dot blot shows relative protein expressions for drivers of cancer aggressiveness and two‐tailed Student's significant test. The western blot pictures are shown in Fig. S3C. (D) Representative TUNEL staining images and quantification from fibrosarcoma tumors derived from MCA‐205 cells (*N* = 4, 32–33 areas per group). The scale bar is 50 μm (E) The immunoblot shows verification of TOMM20 overexpression in MOC‐1 cells. (F) The line graphs show individual tumor volume growth from the initial detection of tumors to the study's endpoint after injection of control EV vs TOMM20 overexpressed MOC‐1 cells into the flank of mice. (G) Tumor volume and significance test using Welch correction. Data are represented as mean ± SE. *P < 0.05, **P < 0.01, and ***P < 0.001 compared to the control group.

To investigate the effect of TOMM20 on tumor size, control EV and TOMM20 overexpressing MCA‐205 cells were injected into C57BL/6 mice (Fig. 3A). We found that mice injected with TOMM20 overexpressing cells had 2.3‐fold higher tumor volume and 1.5‐fold greater tumor weight than control EV cells (Fig. 3B). We verified HA‐TOMM20 overexpression in the tumors using immunoblots from five different tumors in each group (Fig. S5) and found that the driver of cancer invasiveness N‐cadherin, mitochondrial metabolic drivers TIGAR and monocarboxylate transporter (MCT1), antiapoptotic BCL‐2, and proapoptotic BAD were upregulated. Additionally, the ratio of activated caspase 3 to total caspase 3 was downregulated in the TOMM20 overexpressing tumors consistent with reduced apoptosis (Fig. 3C; Fig. S5). However, we did not see any significant changes in MCT4 levels between control EV and TOMM20 overexpressed MCA205 tumors (Fig. S5). Interestingly, programmed cell death ligand 1 (PD‐L1) was upregulated in TOMM20 overexpressed tumors. To further evaluate apoptosis as a mechanism for the difference in tumor growth, we used the terminal deoxynucleotidyl transferase dUTP nick end labeling assay (TUNEL) on tumor sections and found that the tumors in the TOMM20 overexpressing group had 40% lower apoptotic nuclei compared to control tumors (Fig. 3D).

TOMM20 has previously been identified as being highly expressed in epithelial and lymphoid cancers and is a prognostic biomarker [47, 48]. To confirm our *in vivo* tumor study with an epithelial cancer cell line, we generated TOMM20 overexpressing MOC‐1 cells and verified via western blot analysis (Fig. 3E). We injected mice with MOC‐1 with TOMM20 overexpression and control EV cells and found that TOMM20 overexpression significantly increased tumor volume compared to control EV (Fig. 3F,G). To summarize, TOMM20 upregulation increased tumor size, enhanced the expression of drivers of cancer aggressiveness, and reduced tumor apoptosis.

### 3.4 Upregulation of TOMM20 induced resistance to the BCL‐2 inhibitor venetoclax, the mitochondrial complex IV inhibitor ATO, but increases sensitivity to the complex I inhibitor methylglyoxal and increases CD8+ T‐cell tumor recruitment in the context of immune checkpoint blockade PD1

**Fig. 4:**
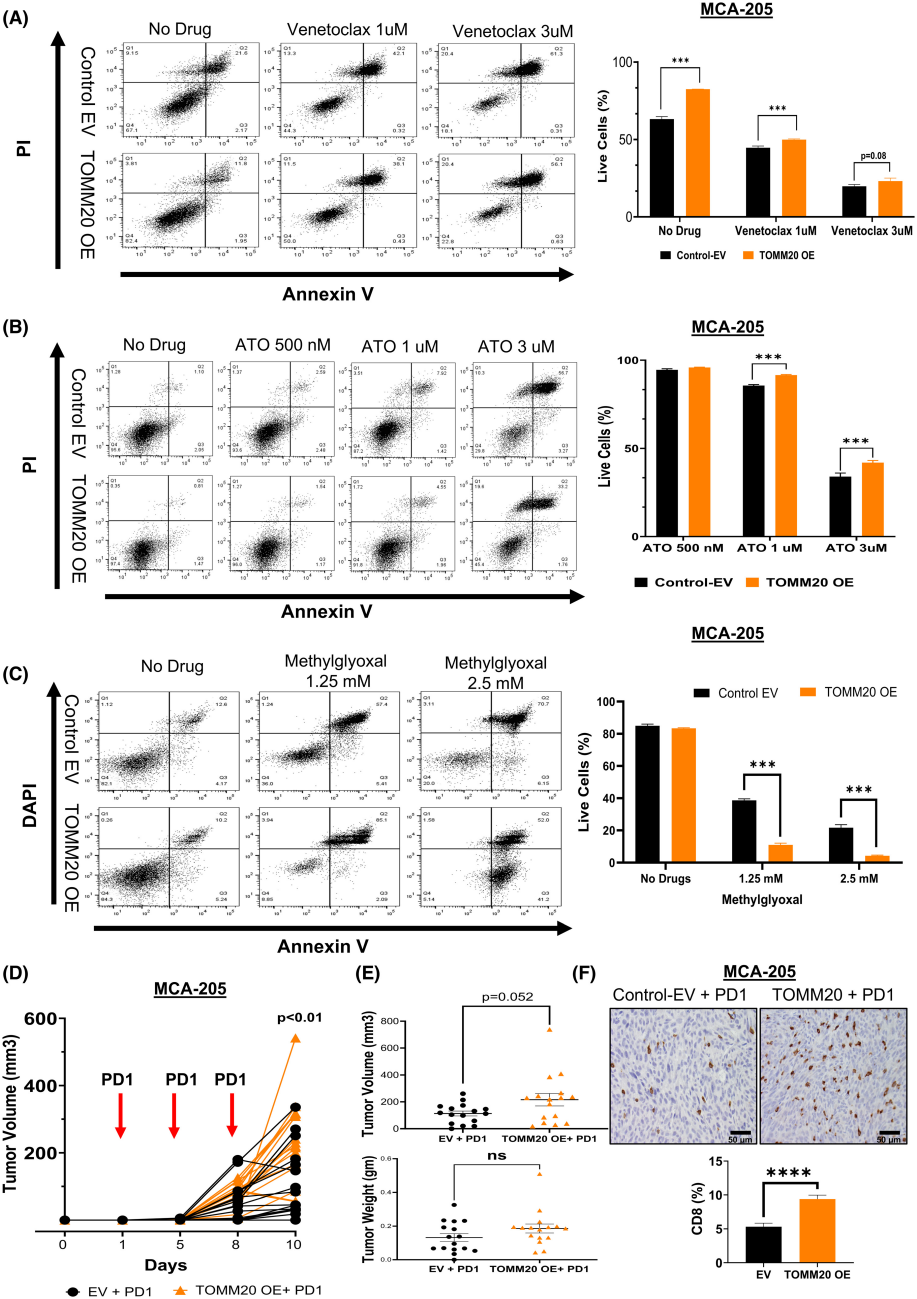
TOMM20 overexpression induces therapy resistance to BCL‐2 inhibitor venetoclax, complex IV inhibitor ATO but sensitive to complex I inhibitor methylglyoxal. (A–C) Live cells were measured using flow cytometry in control EV vs TOMM20 overexpressing MCA‐205 cells (*N* = 4). Cells were stained with APC conjugated with Annexin V and propidium iodide (PI), and apoptotic and live cells were detected. (A) Cells were treated with no drug, 1 μm, and 3 μm ABT‐199 (B) Cells were treated with 500 nm ATO, 1 μm ATO, and 3 μm ATO. (C) Cells were treated with no drug, methylglyoxal 1.25 mm, and methylglyoxal 2.5 mm (*N* = 4). (D) The line graphs show individual tumor volume growth from the initial detection of tumors to the study's endpoint after injection of control EV vs TOMM20 overexpressed MCA‐205 cells into the flank of mice (*N* = 16). The anti‐PD1 antibody was injected on day 1, day 5, and day 8 (red arrows). (E) Tumor volume and weight upon tumor collection. (F) CD8+ staining of the tumor tissues (*N* = 4, 31 areas per group). The scale bar is 50 μm. Two‐tailed Student's t‐test (A–C) and t‐test using Welch correction (D–F); Data represented as mean ± SE. *P < 0.05, **P < 0.01, and ***P < 0.001 compared to the control group.

TOMM20 overexpression in MCA‐205 cells increased antiapoptotic drivers and promoted cell survival, decreased ROS, increased NADH and NADPH levels, and tumor size. We wanted to evaluate whether TOMM20 overexpression also increases resistance to venetoclax (ABT‐199) and arsenic trioxide (ATO), which induce apoptosis by inhibiting BCL‐2 and OXPHOS, respectively [49, 50]. Fibrosarcoma cells were cultured in NuSerum and exposed to vehicle control (DMSO) and different doses of venetoclax to establish the impacts of these drugs on cell death using flow cytometry. Cells overexpressing TOMM20 showed resistance to cell death, with the number of live cells being higher than in the control group without drugs (1.3‐fold), in the presence of venetoclax at 1 μm (1.1‐fold), and venetoclax at 3 μm (1.2‐fold) (Fig. 4A).

Arsenic trioxide (ATO) has been utilized medicinally for thousands of years. It is used to treat acute promyelocytic leukemia (APL) [51, 52, 53]. ATO is a mitochondrial toxin [54] that inhibits OXPHOS complex IV [55] disrupts the mitochondrial membrane potential (MMP) [56], enhances ROS generation, and triggers apoptosis [57]. Hence, we wanted to determine the effects of ATO on cells with TOMM20 overexpression and found that TOMM20 overexpression induced resistance to cell death (Fig. 4B). Specifically, the percentage of live cells increased in the presence of ATO 1 μm (1.1‐fold) and ATO 3 μm (1.2‐fold) with TOMM20 overexpression compared to control cells.

Methylglyoxal (MG) is a highly reactive and toxic dicarbonyl produced mainly as a glycolytic by‐product from dihydroxyacetone phosphate (DHAP) and glyceraldehyde‐3‐phosphate (G3P), though it can also arise from lipid peroxidation and amino acid degradation [58]. Recent studies indicate that MG can inhibit mitochondrial complex I subunits and induce apoptosis in sarcoma, potentially impacting cellular respiration and tumor metabolism. [59, 60]. Cytochrome c also has been shown to protect cells from ROS‐induced damage *in vitro* [61]. We observed that mitochondrial OXPHOS subunit I and OCR were upregulated in TOMM20 overexpressed MCA‐205 cells and downregulated TOMM20 knockdown MCA‐205 and CH2879 cells compared to control cells. Also, recent studies have shown that high OXPHOS pancreatic ductal adenocarcinoma (PDAC) is enriched in mitochondrial respiratory complex I, and treatment with a complex I inhibitor in combination with standard chemotherapy (gemcitabine) had synergistic effects [62]. Mitochondrial transfer in tumors drives anticancer drug resistance [63]. OXPHOS signatures predict resistance to oxaliplatin in colorectal cancer [64]. Also, OXPHOS complex I inhibition has activity in AML models [65]. Methylglyoxal selectively reduced the fraction of live cells in TOMM20 overexpressing compared to control cells with a 3.5‐fold and 5‐fold decrease at 1.25 mm and 2.5 mm methylglyoxal concentrations, respectively (Fig. 4C).

Programmed death‐1 (PD‐1) is a checkpoint protein on T cells that inhibits cytotoxic activity and binds to PD‐L1 in cancer and noncancer cells. Cancer cells expressing high levels of PD‐L1 allow them to evade T‐cell cytotoxicity [41]. Antibodies that target PD1/PD‐L1 binding can enhance the immune response against cancer and are standard‐of‐care therapies in many cancer subtypes. We observed higher expression of PD‐L1 on TOMM20‐overexpressing fibrosarcoma cells (Fig. 1G; Fig. S3C) and tumors (Fig. 3C; Fig. S5). To assess the efficacy of the anti‐PD1 antibody on modulating TOMM20‐overexpressing MCA‐205 tumor growth, we administered three doses of the PD‐1 antibody at three different time points to the mice having tumors with TOMM20‐overexpressing MCA‐205 cells and control EV cells (Fig. 4D). We found that there was a trend toward increased tumor volume with an increase in tumor‐infiltrating CD8+ T cells (Fig. 4D–F).

### 3.5 TOMM20 is necessary for high expression of drivers of cancer aggressiveness, cell growth, and migration in fibrosarcoma cells

**Fig. 5:**
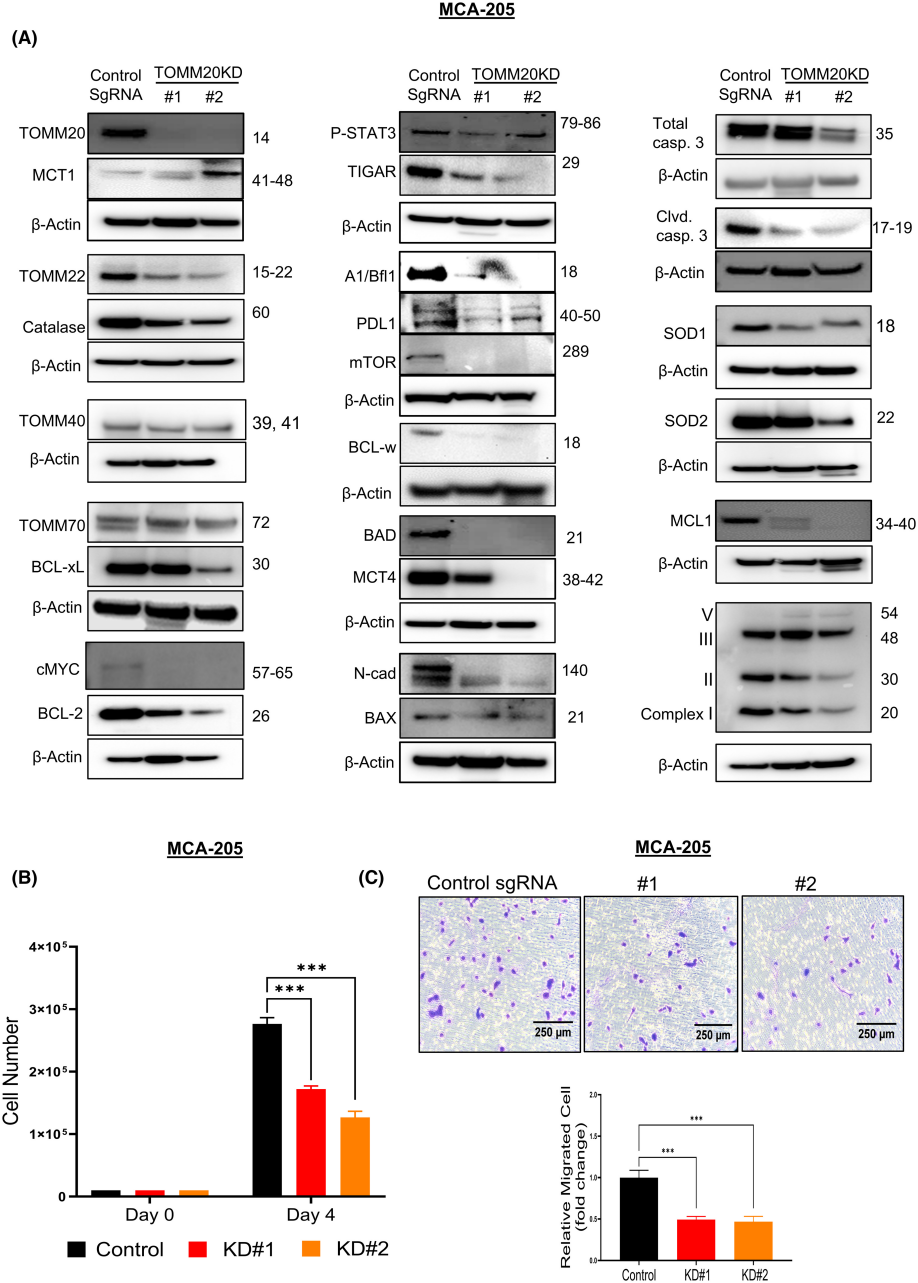
Effect of TOMM20 knockdown on drivers of cancer aggressiveness, cell viability, and migration in MCA‐205 cells. (A) Western blot confirmation of TOMM20 knockdown and the effect of TOMM20 knockdown on drivers of cancer aggressiveness in control sgRNA and two different clones (KD#1 and KD#2) of TOMM20 knockdown MCA‐205 cell. The molecular weight of β‐Actin is 42 KD. Beta‐actin is shown in each blot. (B) Cell viability assay using control sgRNA and two different clones (KD#1 and KD#2) of TOMM20 knockdown MCA‐205 cells (*N* = 4) (C) Cell migration assay using the Boyden chamber in control sgRNA and two different clones (KD#1 and KD#2) of TOMM20 knockdown MCA‐205 cells (*N* = 3). One‐way ANOVA followed by Dunnett's multiple comparisons test among groups (B, C); Data represented as mean ± SE. *P < 0.05, **P < 0.01, and ***P < 0.001 compared to the control group.

We found that TOMM20 overexpression led to increased cancer aggressiveness. Hence, we decided to investigate the impact of knocking down TOMM20 using CRISPR‐Cas9 in sarcoma cell lines and evaluate whether TOMM20 is essential for the expression of drivers of cancer aggressiveness (Fig. 5A; Fig. S6A). Compared to control sgRNA, we found that the expression of invasiveness drivers p‐STAT3 and N‐cadherin levels were downregulated in TOMM20 knockdown MCA‐205 cells (Fig. 5A). Similar to the drivers of invasiveness, the OXPHOS drivers c‐MYC and TIGAR were downregulated in TOMM20 knockdown cells. Surprisingly, all five known BCL‐2 inhibitory proteins (BCL‐2, BCL‐xL, BCL‐w, MCL1, and A1/Bfl1), which are drivers of mitochondrial apoptosis resistance, were downregulated in TOMM20 knockdown cells. Also, we observed that the proapoptotic BH3‐only sensor BAX and BAD were downregulated in TOMM20‐knockdown cells. Both caspase 3 and its activated, cleaved form were downregulated in these cells as well (Fig. 5A).

However, MCT1 was upregulated, and MCT4 was downregulated in TOMM20 knockdown fibrosarcoma cells. Additionally, PD‐L1, mTOR, complex I, complex II, catalase, SOD1, and SOD2 were downregulated in TOMM20 knockdown MCA 205 cells (Fig. 5A).

Next, to assess whether TOMM20 is necessary for cell viability and migration, we examined cell growth and migration of TOMM20 knockdown MCA‐205 cells and control cells and found that cell viability (>30%) (Fig. 5B) and migration (>50%) (Fig. 5C) were inhibited in both clones containing TOMM20 knockdown MCA205 cells. To sum up, TOMM20 is essential for the high expression of proteins that drive cancer aggressiveness, and the knockdown of TOMM20 decreased cell growth and migration in MCA‐205 cells.

### 3.6 Inhibiting TOMM20 reduced OXPHOS in the presence of glutamine, increased NAD+/NADH levels, and increased NADP+/NADPH levels in fibrosarcoma cells

**Fig. 6:**
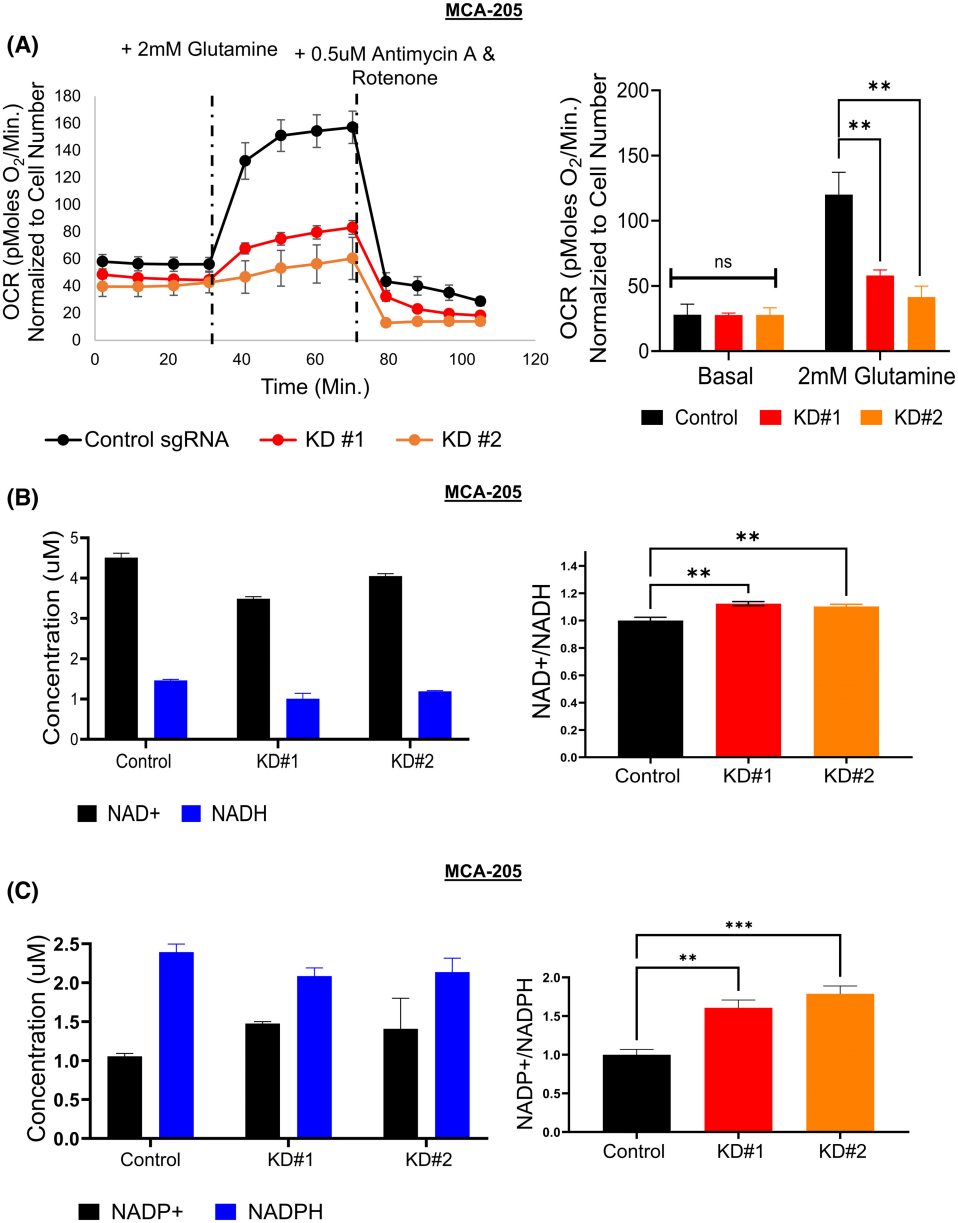
Effect of TOMM20 knockdown on OCR, NADH, and NADPH levels in MCA‐205 cells. (A) OCR was calculated in control sgRNA cells and with TOMM20 knockdown clones. OCR values were normalized to cell number (*N* = 6). Cells were cultured under control media and supplemented with 2 mm glutamine. (B) NAD+ and NADH concentration and ratio in control vs. two different TOMM20 knockdown clones of MCA‐205 cells (*N* = 4) (C) NADP+ and NADPH concentration and ratio in control vs. two different TOMM20 knockdown clones of MCA‐205 (*N* = 4). Two‐tailed Student's *t*‐test (A) and one‐way ANOVA followed by Dunnett's multiple comparisons test among groups (B and C); Data represented as mean ± SE. *P < 0.05, **P < 0.01, and ***P < 0.001 compared to the control group.

Using Mito‐Fuel and Mito‐Stress assays, we observed that TOMM20 overexpressing cells utilized glutamine more efficiently than control cells. In contrast, we observed that knocking down TOMM20 in MCA‐205 cells reduced oxygen consumption by 48% in clone 1 (KD#1) and 34% in clone 2 (KD#2) (Fig. 6A) in the presence of glutamine.

As we had discovered that overexpression of TOMM20 leads to a decreased NAD+/NADH ratio and increased NADPH level; we sought to determine the effects of TOMM20 knockdown. The knockdown of TOMM20 had the opposite effects to overexpression, increasing the NAD+/NADH ratio by more than 10% (Fig. 6B) and the NADP+/NADPH ratio by more than 60% (Fig. 6C) compared to control sgRNA. In sum, suppressing TOMM20 in MCA205 cells reduced OXPHOS in the presence of glutamine while increasing the levels of NAD+/NADH and NADP+/NADPH.

### 3.7 Inhibiting TOMM20 suppressed expression of drivers of cancer aggressiveness, cell growth, and OXPHOS but increased ROS levels in human chondrosarcoma CH2879 cells

**Fig. 7:**
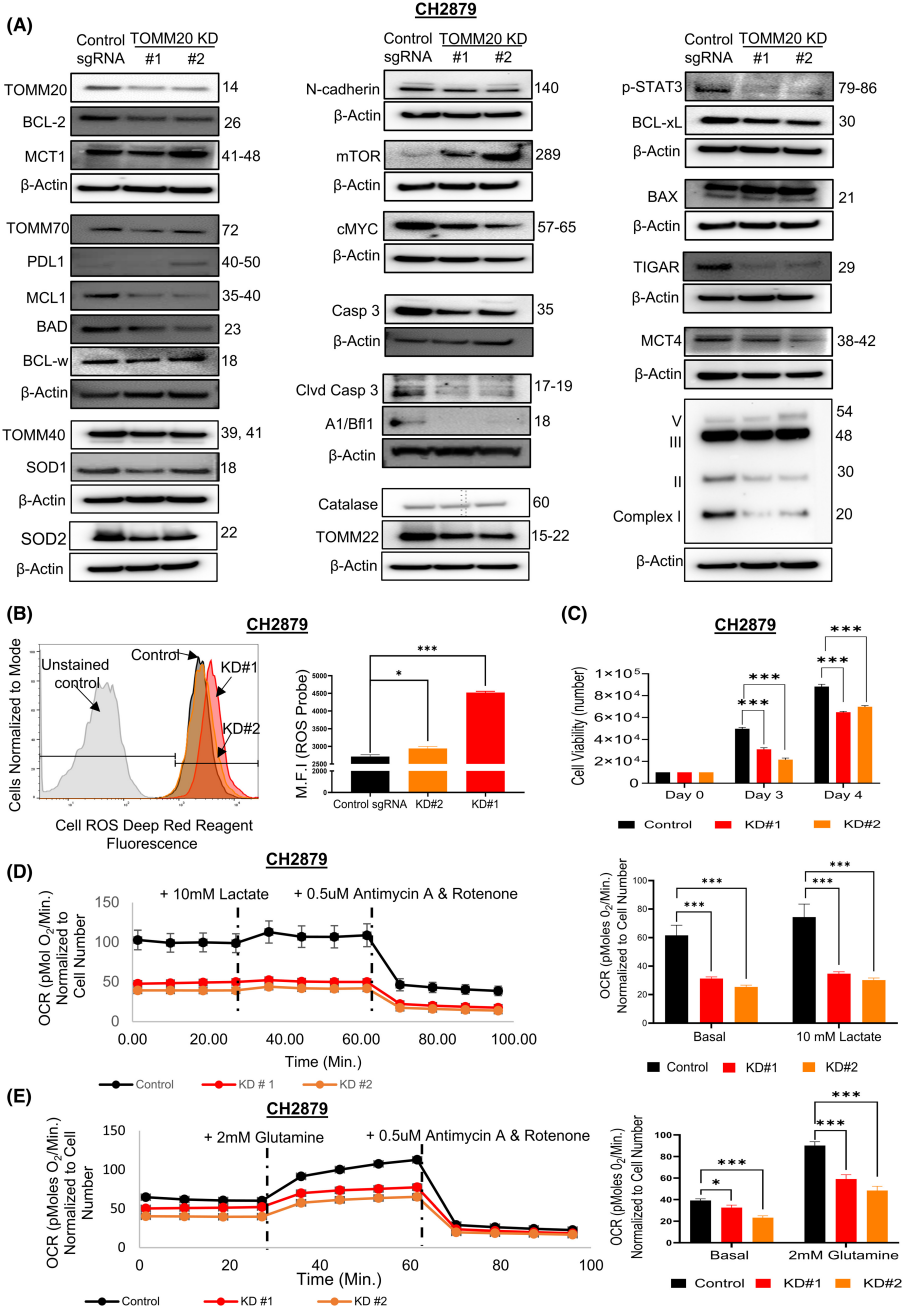
Effect of TOMM20 knockdown on drivers of cancer aggressiveness, cellular ROS levels, cell viability, and OCR in CH2879 cells. (A) Western blot confirmation of TOMM20 knockdown in two different clones (KD#1 and KD#2) CH2879 cells and the effect of TOMM20 knockdowns on the expression of drivers of cancer aggressiveness. The molecular weight of β‐actin is 42 KD. Beta‐actin is shown in each blot. (B) The mean fluorescent intensity (MFI) was measured with flow cytometry using cell ROS deep red reagent in control vs. two clones of TOMM20 knockdown CH2879 cells (*N* = 4) (C) Cell viability assay using control sgRNA and two different clones of TOMM20 knockdown (*N* = 4) (D, E) OCR was calculated in control CH2879 cells (black bar), and with KD#1(red bar) and KD#2 (orange bar), OCR values were normalized to cell number (*N* = 4). Cells were cultured under control media, supplemented with 2 mm glutamine, and control media with 10 mm lactate. One‐way ANOVA followed by Dunnett's multiple comparisons test among groups (B and C) and two‐tailed Student's *t*‐test between groups (D); Data represented as mean ± SE. *P < 0.05, **P < 0.01, and ***P < 0.001 compared to the control group.

We wanted to evaluate if drivers of cancer aggressiveness were downregulated with TOMM20 knockdown in chondrosarcoma. Our laboratory previously showed that drivers of cancer aggressiveness were increased via the overexpression of TOMM20 in CH2879 cells [33]. Hence, we knocked down TOMM20 in CH2879 cells using CRISPR‐Cas9 and found the opposite effect with reduced protein expression with the exception of mTOR, which was increased and no significant change in BCL‐w expression compared to the control sgRNA (Fig. 7A; Fig. S6B).

In addition, using flow cytometry, we found that the knockdown of TOMM20 in CH2879 cells increased mean fluorescent intensity for the oxidative stress fluorescent marker CellROX in both clones; clone 1 increased by 9%, whereas clone 2 increased by 65% compared to control sgRNA (Fig. 7B). The assay for cell viability showed a significant decrease in cell numbers at day 3 and day 4 upon TOMM20 knockdown in both clones (Fig. 7C). Interestingly, the antioxidant enzymes SOD1 and SOD2 also downregulated in the TOMM20 knockdowns clones (Fig. 7A).

Also, to assess OXPHOS, we measured oxygen consumption rates (OCR) using TOMM20 knockdown (KD#1 and KD#2) CH2879 cells and control sgRNA and observed that the knockdown of TOMM20 greatly decreased OCR levels under basal conditions and after the addition of 10 mm lactate (Fig. 7D), or under basal conditions and after the addition of 2 mm glutamine (Fig. 7E). Similar to this, the expression of mitochondrial OXPHOS subunits complex I and complex II was downregulated in the TOMM20 knockdown clones as well (Fig. 7A). To recapitulate, the inhibition of TOMM20 in human chondrosarcoma CH2879 reduced the expression of drivers of cancer aggressiveness, cell viability, and OXPHOS, but increased ROS levels.

### 3.8 Suppression of TOMM20 inhibited tumor growth in xenograft mouse models and decreased oxygen consumption while increasing extracellular acidification in tumor microtissues

**Fig. 8:**
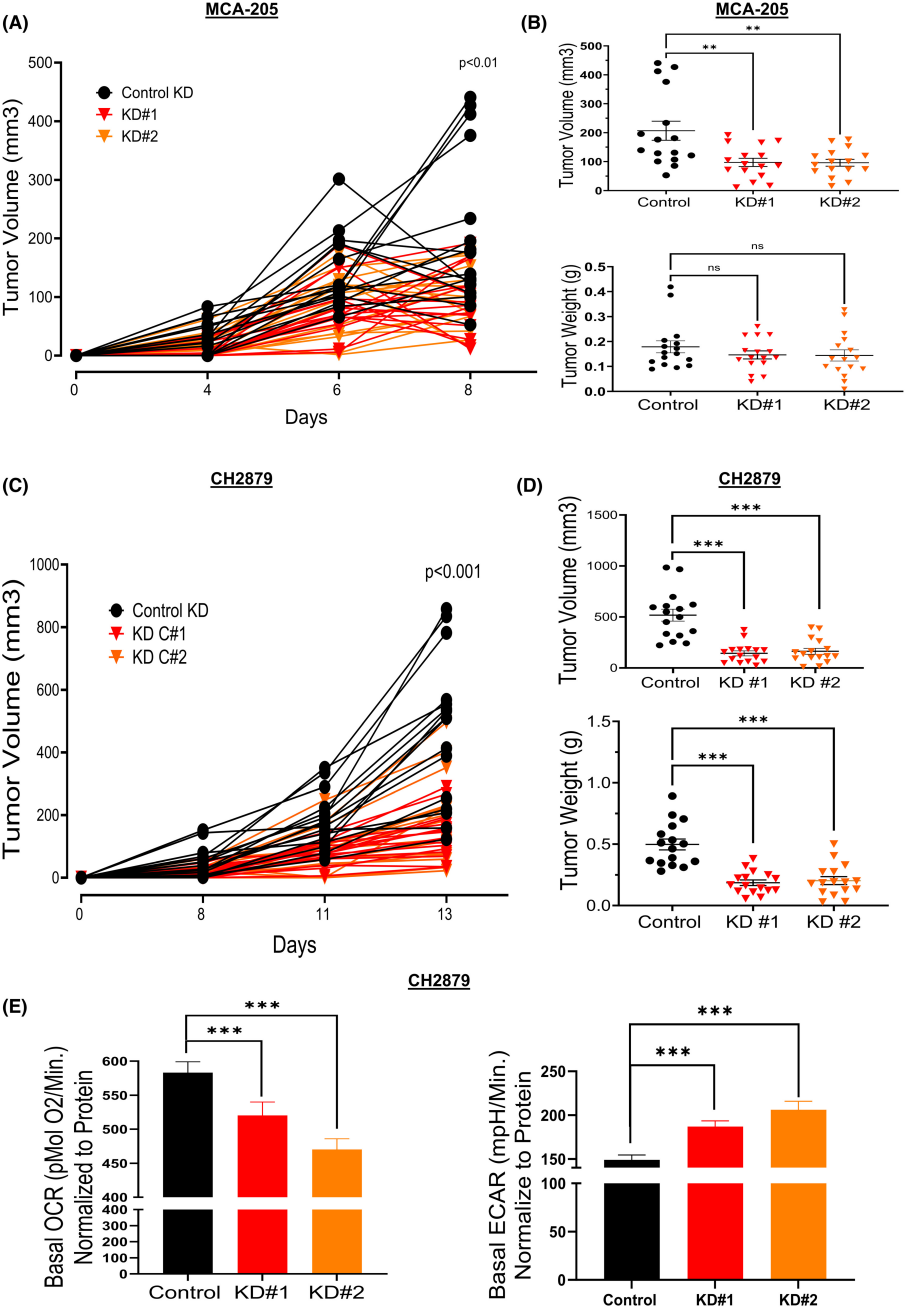
Effects of TOMM20 knockdown on tumor growth and OCR and ECAR in tumor microtissues. (A) The line graphs show individual tumor volume growth from the initial detection of tumors to the study's endpoint in control sgRNA cells (black lines), KD#1 (red lines), and KD#2 (orange lines) in MCA‐205 cells (*N* = 16). (B) Tumor volume and weight upon tumor collection of MCA‐205 control sgRNA (black dots) and TOMM20 knockdowns (red dots and orange dots) (C) The line graphs show individual tumor volume growth from the initial detection of tumors to the study's endpoint in control sgRNA cells (black lines), KD#1 (red lines), and KD#2 (orange lines) in CH2879 cells (*N* = 16). (D) Tumor volume and weight upon tumor collection of CH2879 control sgRNA (black dots) and TOMM20 knockdowns (red dots and orange dots). (E) OCR and ECAR were calculated from tumor microtissue obtained upon the time of tumor collection in control CH2879 tumors (black bar), KD#1 tumors (red bar), and KD#2 tumors (orange bar). Cells were cultured under control media conditions *ex vivo* (*N* = 4). One‐way ANOVA followed by Dunnett's multiple comparisons test among groups (A–D) and two‐tailed Student's *t*‐test between groups (E); Data represented as mean ± SE. *P < 0.05, **P < 0.01, and ***P < 0.001 compared to the control group.

Since TOMM20 suppression reduces the cancer aggressiveness phenotype in MCA‐205 and CH2879 cells, we wanted to establish whether TOMM20 is necessary for tumor growth. Mice receiving MCA‐205 cells with TOMM20 knockdown (Fig. 8A) had more than a 50% reduction in tumor size for both clones (Fig. 8B). The effect of TOMM20 knockdown on tumor size was even greater using CH2879 cells with TOMM20 knockdown (Fig. 8C) resulting in a 70% reduction in tumor size with clone 1 and a 68% reduction with clone 2 (Fig. 8D). We also observed that tumor weight was significantly reduced in the TOMM20 knockdown CH2879 cells compared to controls (Fig. 8D).

**Fig. 9:**
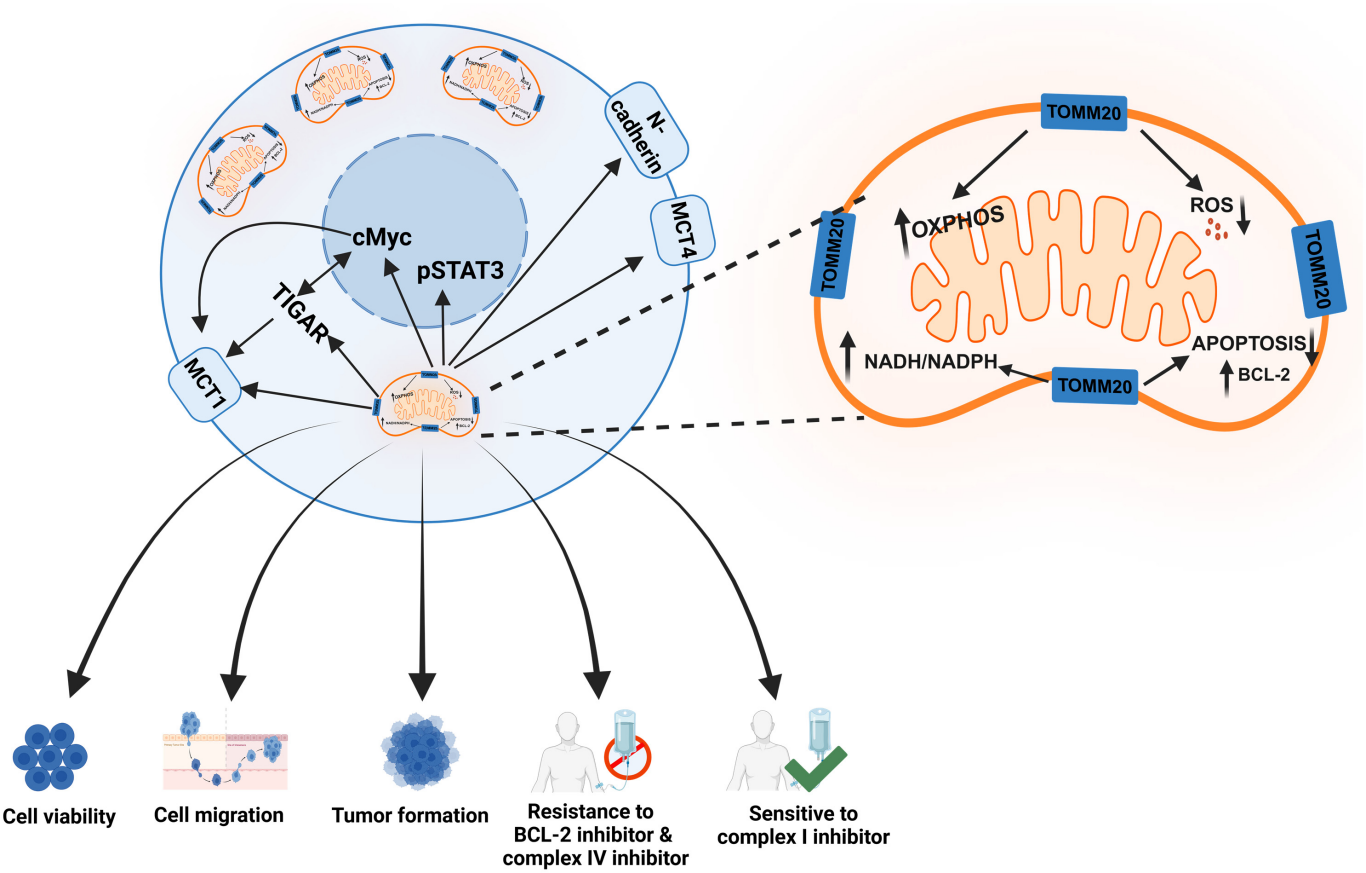
Mechanisms of TOMM20 induce cancer aggressiveness. TOMM20 increases cancer aggressiveness by maintaining a reduced state with increased NADH and NADPH levels, oxidative phosphorylation (OXPHOS), and apoptosis resistance while reducing reactive oxygen species (ROS) levels. Conversely, CRISPR‐Cas9 knockdown of TOMM20 alters these cancer‐aggressive traits.

Tumors were cut, and tumor microtissues were created to determine the OCR and ECAR rates of the tumors from CH2879 TOMM20 knockdown tumors and control sgRNA *ex vivo*. ECAR refers to the decrease in pH of the extracellular environment surrounding cells from tumor microtissues. Baseline OCR was lower in both TOMM20 knockdown clones (KD#1 and #2) compared to control sgRNA (Fig. 8E). Conversely, the baseline ECAR was higher in both clones containing TOMM20 knockdown compared to control sgRNA (Fig. 8E). To summarize, suppressing TOMM20 decreased tumor growth, reduced oxygen consumption, and heightened extracellular acidification in tumor microtissues (Fig. 9).

## 4 Discussion

Mitochondria are essential to the development and progression of cancer, influencing multiple hallmarks of the disease [22, 23]. TOMM20 is essential for the import of nuclear proteins, but its role in driving cancer aggressiveness remains poorly understood. One previous study showed that TOMM20 expression was sufficient to drive tumor growth [33] and other studies have shown that TOMM20 expression is associated with cancer aggressiveness [33, 34, 66, 67]. Here, we investigated the role of TOMM20 in the redox state and oxidative stress, as well as its effects on OXPHOS, cell viability, resistance to apoptosis, and tumor growth by studying fibrosarcoma and chondrosarcoma cells with overexpression or downregulation of TOMM20.

RNA‐seq analysis and gene set enrichment analysis (GSEA) in TOMM20 overexpressing chondrosarcoma L2975 cells revealed suppression of apoptosis, and KEGG pathway analysis highlighted that ferroptosis, ubiquitin‐mediated proteolysis, and necroptosis were suppressed while the ECM‐receptor interaction pathway was activated. Confirming the RNA‐seq insights, we demonstrated that TOMM20 can induce antiapoptotic proteins. Specifically, when TOMM20 is overexpressed in MCA‐205 fibrosarcoma cells, it leads to an upregulation of three members of the apoptosis inhibitory BCL2 family of proteins: BCL‐2, BCL‐xl, and A1/Bfl1. These results are consistent with our lab's previous finding that TOMM20 overexpression in chondrosarcoma cells increased the antiapoptotic protein BCL2 and decreased apoptosis [33]. In addition, TOMM20 downregulation using CRISPR‐Cas9 reduced all known BCL2 apoptosis inhibitory proteins in chondrosarcoma (CH2879) and fibrosarcoma (MCA205). However, we observed that the proapoptotic pore‐forming BAX protein was upregulated in the MCA‐205 cells, and BAD was upregulated in murine tumors obtained from TOMM20 overexpressing MCA205 cells. Although BAX and BAD can induce apoptosis, their activity can be modulated by interactions with antiapoptotic proteins such as BCL‐2 and BCL‐xL [68]. Furthermore, phosphorylation regulates BAD by binding it to 14‐3‐3 proteins, which sequesters BAD in the cytoplasm, preventing it from inducing apoptosis [69]. Although the expression of the activated form of caspase 3 increased in MCA‐205 overexpressing TOMM20 cells and decreased in TOMM20 knockdown CH2879 and MCA‐205 cells, the ratio of cleaved caspase to caspase 3 was decreased in MCA‐205 tumors with TOMM20 overexpression. Also, we found that drivers of cancer cell migration and invasion, such as p‐STAT3 and N‐cadherin, were upregulated with TOMM20 overexpression and downregulated in TOMM20 knockdown cells. The monocarboxylate transporter MCT1 was upregulated in TOMM20 overexpressed MCA‐205 cells and tumors. Similarly, we previously showed that MCT1 was upregulated in aggressive human chondrosarcoma cells [33], and human chondrosarcoma tumors [33], as well as human chordoma tumors [34]. Knockdown of TOMM20 increased MCT1 in MCA‐205, but no significant change was observed in CH2879 cells. Monocarboxylate transporter 4 (MCT4) expression was reduced with TOMM20 downregulation in MCA‐205 and CH2879 cells and increased when TOMM20 was overexpressed in MCA‐205 cells. In addition, OXPHOS drivers c‐MYC and TIGAR were modulated by TOMM20 expression in MCA‐205 and CH2879 cells. Previous studies have shown that lactate uptake and mitochondrial metabolism are upregulated by c‐MYC, MCT1, and TIGAR [33, 36, 70], and lactate export is mediated by MCT4 and is highly expressed in glycolytic cancer cells [71]. We showed for the first time that besides c‐MYC, MCT1, and TIGAR expression, TOMM20 can modulate MCT4 expression. MCT1 and MCT4 induce cell proliferation, migration, and invasion in numerous cancers [72, 73]. TOMM20 overexpressing cells have higher maximum reserve capacity and can use lactate and glutamine as substrates for their increased OCR highlighting how cancer cells have high metabolic flexibility. This metabolic flexibility has been shown to support the survival and growth of cancer cells under metabolic stress. Also, we demonstrate that TOMM20 can promote cell viability and migration in MCA‐205 and CH2879 cells.

Mitochondria play an essential role in producing and regulating ROS, which is vital for cellular signaling and contributes to the development of various diseases, including cancer. ROS has different functions, acting as signaling molecules that promote cell proliferation at low to moderate levels; while at high levels, they can become cytotoxic, overwhelming the cell's antioxidant defenses and causing oxidative damage [74]. There are no studies to our knowledge investigating the role of TOMM20 on ROS generation. However, one study indirectly shows that degradation of TOMM20 by androgen receptor antagonists leads to increased ROS production in prostate cancer cells, contributing to drug resistance [75]. Using MCA‐205 and CH2879, we demonstrate that overexpression of TOMM20 decreases ROS production and increases antioxidant enzymes catalase, SOD1, and SOD2, and knocking down TOMM20 increases ROS production while decreasing the antioxidant enzymes SOD1 and SOD2. Modulating ROS as an anticancer strategy is promising [76]. Also, we observed that TOMM20 can modulate complex I OXPHOS subunit expression, and targeting complex I increased apoptosis in the TOMM20 overexpressing cells compared to control cells. Future studies will need to determine if OXPHOS mediates the effects of TOMM20 on ROS.

Previously, our research showed that TOMM20 overexpression increased OXPHOS in chondrosarcoma. OXPHOS was increased in the presence of glucose and lactate [33]. Here, we demonstrate that TOMM20 induces OXPHOS under basal conditions, in the presence of lactate, and in the presence of glutamine in fibrosarcoma and chondrosarcoma and that TOMM20 knockdown reduces the OCR in all conditions. Furthermore, we found that TOMM20 overexpression boosts the cells' maximum reserve capacity, underscoring its role in enhancing mitochondrial function. Notably, in chondrosarcoma tumors with TOMM20 knockdown, oxygen consumption was decreased, while extracellular acidification rates (ECAR) were elevated, suggesting a metabolic shift away from OXPHOS.

Oxygen consumption and mitochondrial metabolism require an abundant and continuous supply of reducing agents such as NADH [46] and NADPH [45]. NADH is essential for driving OXPHOS, while NADPH is crucial for antioxidant defense and biosynthetic reactions. Here, we demonstrate that TOMM20 overexpression increases NADH and NADPH levels, while TOMM20 knockdown has the opposite effect, increasing the levels of the oxidized forms with increased NAD+/NADH and NADP+/NADPH ratios. Future studies will need to determine if reduced NADH and NADPH are required for OXPHOS induced by TOMM20.

Sarcomas are frequently resistant to standard chemotherapy agents [7, 10, 33, 77]. We demonstrated that TOMM20 overexpressing cells induce drivers of apoptosis resistance, and these drivers are decreased in TOMM20 knockdown cells. In addition, there was a reduction in apoptosis in TOMM20 overexpressed MCA205 tumors.

Hence, we targeted apoptosis pathways using the FDA‐approved mitochondrial targeting drugs venetoclax and arsenic trioxide (ATO), which inhibit BCL‐2 and OXPHOS, respectively [56]. We also used methylglyoxal to inhibit the mitochondrial complex I respiratory chain. We show that venetoclax and ATO fail to induce apoptosis while methylglyoxal significantly increased vulnerabilities to apoptosis in TOMM20 overexpressing MCA‐205 cells compared to control cells. Therefore, we have demonstrated that TOMM20 in fibrosarcoma induces resistance to BCL‐2 inhibitor, and immune checkpoint inhibition but is sensitive to complex I inhibitor. Future studies are necessary to determine whether the increased vulnerability to apoptosis caused by complex I inhibitors is due to elevated ROS levels or alternative mechanisms. This research underscores the potential of targeting mitochondrial vulnerabilities in TOMM20‐overexpressing tumors.

TOMM20 can modify cell growth and migration in MCA‐205 and CH2879 cells *in vitro*. *In vivo*, we showed that TOMM20 overexpression led to larger tumors, while TOMM20 knockdown reduced tumor size. Hence, we showed that the expression of TOMM20 can modulate mouse fibrosarcoma and human chondrosarcoma growth. Future studies are necessary to confirm these results with human fibrosarcoma cells.

Our study aligns with previous research, which has found links between TOMM20 expression and cancer progression in humans [33, 34, 67, 78]. Importantly, we identified a vulnerability in TOMM20‐overexpressing cells to complex I inhibitors, emphasizing a promising therapeutic avenue. Our findings indicate that TOMM20‐overexpressing cells are particularly sensitive to complex I inhibitor, which significantly increase apoptotic vulnerability. Future studies are warranted to investigate whether this heightened sensitivity is mediated through elevated ROS levels or other underlying mechanisms. It is possible that the molecular mechanisms modulating tumor growth with TOMM20 overexpression and downregulation are distinct, for example, proteostasis imbalance and activation of stress response pathways might be important with overexpression, while downregulation affects mitochondrial import, as shown for TOMM22 [79] and will need to be studied in the future. Additionally, further research is needed to explore whether targeting TOMM20 expression in tumors could effectively reduce cancer aggressiveness and suppress tumor growth.

## 5 Conclusions

TOMM20 induces sarcoma aggressiveness by increasing oxidative phosphorylation (OXPHOS), maintaining a reduced redox state, decreasing ROS, increasing migration, and enhancing resistance to apoptosis. Its overexpression promotes *in vivo* tumor growth while increasing sensitivity to an OXPHOS complex I inhibitor. Conversely, TOMM20 knockdown suppresses these effects, reducing tumor progression and OXPHOS *in vivo*. These findings highlight TOMM20 as a potential therapeutic target.

## Conflict of interest

All authors have read and approved of its submission to this journal. The authors declare that they have no conflict of interest.

## Author contributions

RI: Conceptualization, methodology, investigation, validation, formal analysis, resources, writing—original draft, writing—review and editing, visualization. MER: Conceptualization, methodology, investigation, validation, formal analysis, resources, writing—review and editing, visualization. ZL: Conceptualization, methodology, investigation, formal analysis, validation, resources. DWM: Conceptualization, methodology, investigation, formal analysis, validation, resources, writing—review and editing. VDB: Conceptualization, methodology, investigation, formal analysis, validation, resources, writing—review and editing. ES: conceptualization, methodology, investigation, formal analysis. MPMC: Conceptualization, supervision, writing—review and editing. NJP: Conceptualization, supervision, writing—review and editing. ABM: Conceptualization, supervision, project administration, writing—review and editing, funding acquisition. UMO: Conceptualization, supervision, project administration, writing—original draft, writing—review and editing, funding acquisition.

## Peer review

The peer review history for this article is available at https://www.webofscience.com/api/gateway/wos/peer‐review/10.1002/1878‐0261.70002.

## Supporting information


**Fig. S1.** Impact of TOMM20 on Reactome pathways. (A) TOMM20 expression levels using RNA‐seq data. (B) Gene set enrichment analysis (GSEA) of Reactome pathways highlighting TOMM20 roles in apoptosis‐related pathways.

**Fig. S2.** Effects of TOMM20 on cancer gene set enrichment pathways. (A–D) RNA‐seq data for L2975 TOMM20 versus control EV. Pathway schematics of the KEGG pathways, including (A) ferroptosis, (B) necroptosis, and (C) ubiquitin‐mediated proteolysis. (D) Pathway enrichment analysis from Gene set enrichment analysis (GSEA). NES, normalized enrichment score. *P* < 0.05 was considered statistically significant.

**Fig. S3.** The effect of TOMM20 on markers of cancer aggressiveness in MCA‐205 cells. (A) The immunofluorescence figure (left) shows mitochondria in MCA‐205 cells with the mitochondrial marker HSP60 (green) and HA‐tagged TOMM20 (red). The colocalization of mitochondria and HA‐tagged TOMM20 is shown in yellow. The scale bar is 50 μm. Quantification of the fluorescence signal is shown on the right (*N* = 3) (B) Western blots show the total protein, HA expression, and β‐actin expression in control EV and TOMM20 overexpressed MCA‐205 cells (*N* = 5). (C) The western blots show the verification of TOMM20 overexpression and its effect on different markers of cancer aggressiveness in control EV and TOMM20 overexpressed MCA‐205. Our TOMM20 overexpression MCA205 has an endogenous TOMM20 band (lower big band) and an HA‐tagged TOMM20 band (upper narrow band). The molecular weight of β‐Actin is 42 KD. Beta‐actin is shown in each blot. Student's *t*‐test between groups (A–C); Data are represented as mean ± SE. *P < 0.05, **P < 0.01, and ***P < 0.001 compared to the control group.

**Fig. S4.** TOMM20 effect on mitochondrial maximum reserve capacity, fuel oxidation, and NAD+/NADH, and NADP+/NADPH ratio in MCA‐205 cells. (A) OCR was calculated in control (black bar), and TOMM20 overexpressed (orange bar) MCA‐205 and CH2879 cells after using 2 μM FCCP, OCR values were normalized to the cell number (*N* = 6). (B) Mito‐Fuel assay of TOMM20 overexpressed and control EV cells. The experiment used a Seahorse Mito‐Fuel kit in an XFe96 extracellular flux analyzer, as detailed in the materials and methods. The dependency, flexibility, and capacity percentage were calculated for fuel glutamine, glucose, and fatty acid. (C) NAD+/NADH ratio in control vs TOMM20 overexpression MCA‐205 cells. Fold change normalized with control EV. (D) NADP+/NADPH ratio in control vs TOMM20 overexpression MCA‐205 cells. Fold change normalized with control EV. Student's *t*‐test between groups; Data are represented as mean ± SE.

**Fig. S5.** The effect of TOMM20 on drivers of cancer aggressiveness in MCA‐205 tumors. Tissues from five different tumors from the control EV and TOMM20 overexpressed group collected and run western blot show expression of the markers of cancer aggressiveness. TOMM20 overexpression is determined by HA antibody as our plasmid has HA‐tagged TOMM20. The molecular weight of β‐Actin is 42 KD.

**Fig. S6.** The western blots show the verification of β‐actin expression and total protein expression ratio in control sgRNA and TOMM20 KD cells. (A) MCA‐205 (*N* = 6) and (B) CH2879 cells (*N* = 6). One‐way ANOVA followed by Dunnett's multiple comparisons test among groups; Data are represented as mean ± SE.

## Data Availability

Some of the data supporting this study's findings can be found in Raw Fig. 5 and Fig. S5 in the supporting document of this article. Additionally, the RNA‐seq dataset output is provided in the supporting document section. All data underlying this study's findings are available from the corresponding author (Ubaldo Martinez‐Outschoorn, ubaldo.martinez‐outschoorn@jefferson.edu) upon reasonable request.
